# Measurement of Social Strain in People with Dementia: A Preliminary Study of the Reliability and Validity of the Negative Relationship Quality Questionnaire in Indonesia

**DOI:** 10.3390/geriatrics7050099

**Published:** 2022-09-15

**Authors:** Amelia Nur Vidyanti, Galenisa Falinda Santika Putri, Aditya Rifqi Fauzi, Rizqa Nafiati, Astuti Prodjohardjono, Christantie Effendy

**Affiliations:** 1 Department of Neurology, Faculty of Medicine, Public Health and Nursing, Universitas Gadjah Mada, Yogyakarta 55281, Indonesia; 2 Department of Neurology, Dr. Sardjito General Hospital, Yogyakarta 55281, Indonesia; 3 Department of Medical-Surgical Nursing, Faculty of Medicine, Public Health and Nursing, Universitas Gadjah Mada, Yogyakarta 55281, Indonesia

**Keywords:** dementia, social strain, NRQ, negative relationship quality, validity study, reliability study

## Abstract

People with dementia (PWD) may exhibit symptoms that negatively affect their relationships with their families or friends which could cause social strain. The Negative Relationship Quality (NRQ) questionnaire can be used to measure social strain in PWD. There has never been an Indonesian adaptation of the NRQ. This preliminary study aimed to measure the validity and reliability of the NRQ among PWD in Indonesia (NRQ-INA). This study used a cross-sectional design. Forward–backward translation methods were conducted first. Pearson’s correlation and factor analysis were employed for the validity test. Cronbach’s alpha and test–retest were used to determine reliability. The NRQ-INA has four parallel items related to social strain that are divided into three subscales and asked to spouse/partner, family members, and friends, leading to a total of 12 questions. The results of validity testing from 60 respondents showed that all items in the NRQ-INA were strongly valid with correlation coefficients (*r*) of >0.8 (*p* < 0.01). Factor analysis showed a convergence with the variance explained of more than 50% for all items in each subscale, which also indicated that NRQ-INA had acceptable construct validity to measure social strain. Cronbach’s alpha values (α) were 0.926, 0.942, and 0.938 for the subscales of spouse, friends, and family members, respectively. The correlations of test–retest reliability for all items were >0.7 (*p* < 0.01), demonstrating a reliable NRQ-INA measurement. In conclusion, NRQ-INA had a good validity and reliability to measure social strain in PWD. Further study of the concurrent validity among PWD is still needed.

## 1. Introduction

Dementia is a prevalent degenerative illness that primarily affects the elderly [1]. The most common symptom of dementia is impaired memory function, particularly impairment in delayed recall and episodic memory [2]. Personality and behavioral changes, apathy, social isolation, and communication problems are also symptoms of dementia which are associated with high levels of distress for patients and caregivers [3].

According to the World Health Organization (WHO), around 50 million individuals worldwide have dementia, with approximately 10 million new cases diagnosed each year. The number of people with dementia (PWD) is expected to reach 82 million in 2030 and 152 million in 2050 [4]. The incidence of dementia in Indonesia is still unknown due to a lack of data. According to Alzheimer’s Disease International (ADI), dementia affects around 40% of the population in Indonesia, Thailand, and Sri Lanka above the age of 65 [5]. Moreover, there were 1,033,000 PWD in Indonesia in 2015, with a predicted increase to 1,894,000 by 2030 [5].

The European INTERDEM network’s Social Health Taskforce created the notion of social health in regards to dementia. Social health is the relational domain of health as defined by the World Health Organization (WHO) in 1946, alongside physical and psychological health [6]. Social health goes beyond pathology and connects social life with health [7]. There are several social factors which can influence the outcomes of dementia, such as level of education, social isolation (loneliness), leisure activities, social engagement, and environmental support [7,8,9,10].

Social engagement and environmental support may positively impact the social health of persons with dementia [11]. Social health is critical for persons with dementia and their families, who often provide care at home and experience substantial challenges while doing so [12]. Furthermore, epidemiological research found that people with dementia (PWD) who were more likely to have inadequate social support leading to social isolation and loneliness, may experience an impedance in their cognitive performance and a decrease in their quality of life [13,14,15].

In addition, PWD who mostly rely on others will have restricted activity, limiting their social relationships [16]. The restricted activities are due to the limitations of their physical and cognitive abilities, and these may lead to higher risk of frustration, anger, and agitation in PWD [17,18]. Unfortunately, research investigating the scope and prevalence of social strain among PWD and the how it influences the outcomes of dementia is still limited. This could be attributable to the difficulties in applying appropriate tools to measure social strain in PWD.

Meanwhile, negative relationship quality is a strong predictor of relationship strain [19]. Negative relationship quality refers to individuals’ opinions on how much their social companions irritate them and make too many demands [20]. The Negative Relationship Quality (NRQ) questionnaire developed by Walen and Lachman in 2000 assesses a person’s negative relationships with their immediate surroundings, such as their spouse, friends, and family members [21]. In other words, it can also be used for measuring social strain in PWD.

Due to the importance of measuring social strain for evaluating dementia outcomes and the fact that research related to this issue is still limited, the present study aimed to assess the validity and reliability of the NRQ as a tool to measure social strain among PWD in Indonesia. The NRQ has never been translated into the Indonesian language.

## 2. Material and Methods

This cross-sectional research was conducted to test the validity and reliability of the NRQ questionnaire utilizing the WHO transcultural standards. The questionnaire was the NRQ Indonesian version (NRQ-INA), which consisted of 4 parallel questions to examine the negative relationships of PWD with their caregivers, namely, their spouses, friends, or family members. Due to the fact that this questionnaire has 3 subscales (spouse, friend, family member), the total number of items in this questionnaire is 12 questions. All subjects gave their informed consent for inclusion before participating in the study. This study was approved by the Institutional Review Board of the Faculty of Medicine, Public Health and Nursing at Universitas Gadjah Mada/Dr. Sardjito General Hospital (KE/FK/0105/EC/2020).

### 2.1. Participants

The participants in this research were the outpatient PWD at the Memory Clinic, Dr. Sardjito General Hospital Yogyakarta from January 2020 to April 2021. We included individuals who match the following requirements: (1) People diagnosed with mild to moderate dementia, (2) who can communicate in Indonesian. The exclusion criteria were as follows: (1) aphasia, (2) severe dementia, (3) lack of a caretaker residing in the same residence, and (4) inability to do a follow-up at the Memory Clinic, Dr. Sardjito Hospital Yogyakarta.

We diagnosed the patients with dementia based on Diagnostic and Statistical Manual of Mental Disorders V [22], leading to diagnosis of dementia based on the etiological causes, i.e., Alzheimer’s disease, cerebrovascular (vascular) lesion, mixed-type, or other causes such as space-occupying process (SOP), traumatic brain injury, epilepsy, etc. The severity of dementia was defined by using the Clinical Dementia Rating (CDR) scale [23] and the Montreal Cognitive Assessment (MoCA) [24]. We excluded patients with severe dementia if the CDR scale was 3 [23] and MoCA score was <12 [25]. Patients with inability to comprehend the questions or commands given and/or inability to speak, namely aphasia, were also excluded.

A sample is part of the total objects of study gathered and represents the entire population [26]. Samples were acquired using a non-probability sampling approach employing convenience sampling. The number of samples were determined from 5 or 10 multiplied by the number of questions or indicators [27]. Based on this method, the sample employed in this research was 60, where 12 indicators of the NRQ-INA inquiry were multiplied by 5.

Initially we collected 63 respondents. There were 3 patients who were excluded due to inability to comprehend some questions. Before we excluded them, we had asked the caregiver to corroborate the information for them. However, the respondents were still unable to comprehend some questions, requiring them to be excluded. The total number of respondents who participated in the final analysis were 60 patients.

### 2.2. NRQ Measurement

This research investigated the reliability and validity of the NRQ-INA according to the WHO transcultural standards. First, the NRQ translation was done by the Language Institute of Universitas Gadjah Mada. Furthermore, an evaluation of the content and structure of the NRQ-INA concept was conducted by the Neurobehavior Team of the Department of Neurology, Faculty of Medicine, Public Health, and Nursing, Universitas Gadjah Mada. The following relationships were examined in the NRQ [21]: spouse/partner, friends, and family members (excluding one’s spouse or partner).

Strained network exchanges were quantified using four parallel questions/items, which read as follows [21]: (i) How often do they criticize you? (ii) How often do they make too many demands on you? (iii) How often do they let you down when you are counting on them? and (iv) How often do they get on your nerves? All questions/items were graded on a 4-point Likert scale (1 = often, 2 = sometimes, 3 = seldom, 4 = never). Items were recoded such that lower scores indicated greater strain.

### 2.3. NRQ Translation

The translation procedure was tailored to international standards [28]. The following phases of language transmission were recommended for inclusion in the study:

#### 2.3.1. Forward Translation

Two people translated the NRQ questionnaire for this research. The first translator was a health specialist familiar with the terminology included in the NRQ questionnaire and understood the instrument’s culture, original language (English), and the target language (Indonesian language/Bahasa Indonesia). This first translation was completed by translators with a health-professional background who had lived in English-speaking countries and had experience writing surveys but whose native language is Bahasa Indonesia. The second translation was completed by a translator who had lived in English-speaking countries but was not a medical professional.

#### 2.3.2. Expert Panel

This procedure was conducted to discuss the results of the forward translation.

#### 2.3.3. Backward Translation

The NRQ questionnaire was re-translated from Bahasa Indonesia back into the instrument’s original language as part of this operation (English). Two translators conducted the backwards translation, one from the observed instrument area and one from a different region (sworn).

#### 2.3.4. Expert Committee

Expert groups examined the results of the forward and reverse translations. The final translated form of the questionnaire was semantically, idiomatically, and conceptually corrected according to two translators, two pharmacists, and one clinician.

#### 2.3.5. The Final Version of Validity and Reliability

The final version of the translation was assessed for validity and reliability. In this experiment, construct validity testing was employed. In addition, internal consistency testing and test–retest reliability were utilized to confirm and validate the dependability.

#### 2.3.6. Validity Test

Validity research was done to test the validity of the questionnaire. A questionnaire is deemed legitimate if the questions on the questionnaire can disclose anything that will be assessed by the questionnaire [27]. This research linked the scores of each item with the overall score and then processed them using SPSS for Windows (IBM Corp., Armonk, NY, USA). All analyses were performed with a significance level α = 0.05.

The validity test was computed using Pearson’s correlation coefficient (*r*). The criteria of interpreting a validity coefficient are as follows [29]: very beneficial or strongly valid (*r* > 0.35), likely to be useful (*r* = 0.21–0.35), depends on circumstances (*r =* 0.11–0.20), and unlikely to be useful (*r* < 0.11). The construct validity was further analyzed by using factor analysis (principal component analysis) to investigate whether this questionnaire measures social strain in PWD in the same way as the original English version.

#### 2.3.7. Reliability Test

The reliability study examined the consistency of the questionnaire. A questionnaire is reliable if the responses to the questions are consistent or constant over time [27]. In this research, the reliability test was conducted by using two methods: internal consistency and test–retest reliability.

Cronbach’s alpha coefficients (α) were measured to determine the internal consistency of reliability. The criteria of interpreting an internal consistency reliability coefficient are as follows [29]: excellent (α ≥ 0.90), good (α = 0.80–0.90), adequate (α = 0.70–0.79), and less applicable (α < 0.70).

The test–retest reliability study was conducted on the same respondent 1 week after the first test was delivered. Pearson’s correlation coefficient was used to assess the level of acceptance between test and retest. All analyses were performed with a significance level α = 0.05.

## 3. Results

A total of 60 PWDs were recruited for this investigation. Males outnumbered females, with 39 (65%) males and 21 (35%) females for a ratio of 1.85:1. Dementia was classified according to age into two categories: senile dementia (over 65) and presenile dementia (less than 65 years of age). Sixty-seven percent of the individuals in this research were over the age of 65, while twenty patients were under 65 (33.3%).

The number of patients with vascular dementia was 35 (58.3%), Alzheimer’s dementia was 12 (20%), mixed-type dementia was 9 (15%), and other dementia was 4 (6.7%). Other dementias include those caused by specific disorders, such as space-occupying process (SOP)-related dementia, post-traumatic brain injury (TBI)-related dementia, subcortical dementia, and epilepsy-related dementia. Table 1 summarizes the demographic characteristics of the respondents in this research.

The results of our validity study showed that NRQ-INA had a strong validity (*r* > 0.35). The findings for each item were summarized in Table 2 below:

Further one-factor CFA using principal component analysis showed that Kaiser–Meyer–Olkin (KMO) test yielded an index of 0.840, 0.859, and 0.826 for the subscales of spouse, friend, and family member, respectively (Table 3). These results suggested support for factor analysis. A principal component analysis showed a one-factor solution (convergence with the variance explained was more than 50%) that also provided support for the construct validity of the NRQ-INA questionnaire to measure social strain.

For internal consistency reliability, it was found that the Cronbach’s alphas of the NRQ-INA questionnaire for each subscale were as follows: spouse (α = 0.926), friend (α = 0.942), and family member (α = 0.938). The Cronbach’s alpha values for each item of the questionnaire are shown in Table 4.

In addition to internal consistency reliability, we also measured test–retest reliability to assess the external consistency. The results of the test–retest reliability from the first and second reliability tests showed good and positive correlations (*p* < 0.001). This indicate that the questionnaire was reliable (Table 5).

The NRQ-INA questionnaire was valid and reliable. Subsequently, we found that the median score of the NRQ-INA for all subscales was quite high based on gender, age, and dementia type. This finding indicated that social strain in dementia patients was quite low, although it was not significant (Table 6).

## 4. Discussion

In our study, the NRQ-INA was valid and reliable to measure social strain in people with dementia. To our knowledge, this is the first study to measure the validity and reliability of this questionnaire in regards to dementia.

There is scarce research measuring the validity and reliability of NRQ. The original English version of the NRQ was developed by Walen and Lachman (2000) [21]. They measured the costs and benefits of social support and social strain from the partner, family, and friends of healthy adult people. Although they did not measure the validity of the NRQ questionnaire, they found that social strain was positively correlated with negative mood, health problems, and life dissatisfaction [21].

Other studies conducted by Birditt, et al. (2009) [20] and Akiyama, et al. (2003) [30] had similar results. Both studies used a modified NRQ questionnaire. They did not mention the validity results of the questionnaire. However, they reported that negative relationship quality (as measured by the NRQ questionnaire) decreased over time among people with different relationship (friends or family member), but increased over time with the partner/spouse [20,30]. This could indicate that the NRQ may be valid to measure the level of social strain.

For the reliability of this questionnaire, previous studies found that NRQ had acceptable reliability with Cronbach’s alphas of 0.81 (spouse/partner strain), 0.79 (friend strain), and 0.80 (family strain) [21]. Our findings further support this research. Another study corroborated our findings; they found that the Cronbach’s alphas of the NRQ were 0.82 (spouse), 0.81 (friend), and 0.79 (family member) for Japanese healthy adult people (30). All these results indicate that the NRQ has good reliability. Nevertheless, another study found different results; the NRQ had poor reliability (less applicable) when measuring negative relationship quality with Cronbach’s alpha values of 0.60 (spouse), 0.51 (friend), and 0.69 (family member) [20]. The poor reliability found in this prior study may be due to a small number of available items (a small number of participants who reported that their friend or partner made too many demands of them) [20].

Negative relationship quality is mostly studied in social sciences. There are no prior studies demonstrating this issue as a predictor of poor outcomes among dementia patients.

Our study contributes to providing further evidence of the validity and reliability of NRQ-INA as a valid and reliable tool to measure social strain among PWD. In addition, although the present study is still a preliminary study, we also demonstrated that vascular dementia was the most prevalent type of dementia in Indonesia, not Alzheimer’s disease as reported by other studies [31,32]. This is assumed to be owing to disparities in exposure to cerebrovascular risk factors such as hypertension, smoking, obesity, and diabetes mellitus [33] in Indonesia. In terms of gender, males are more likely than females to develop vascular dementia [31]. This was comparable to our findings, in which there were more males than females who had vascular dementia.

The present study also reported that the level of social strain among PWD was quite low although this was not significant. Studies of social networks usually establish a correlation between social support and greater psychological well-being and physical health, demonstrating generally favorable impacts [21]. However, great progress has been made in understanding the processes involved in social interactions and their consequences on well-being [21]. While numerous researchers have started to study social strain, research is still equivocal as to whether strain has greater, comparable, or lesser impacts than support [21]. Therefore, the balance and interaction between social support and social strain may influence the outcome of whether the patients will have a greater social strain or a lower one. Similar research utilizing a different questionnaire from prior studies discovered that this kind of survey was valuable in analyzing both positive and negative features of pregnant and parenting teenage mothers [34], as well as children with serious emotional disturbance [35] within social support networks.

This research does have some limitations. The findings of this research may not apply to a large population due to the relatively small sample size; hence, further research with a bigger sample size is required. Second, patients with severe dementia were excluded which may limit the general application of the questionnaire to all stage of dementia. Third, the prevalence of social strain among PWD and the relationship between social strain and dementia could not be addressed in the present study. Finally, the concurrent validity as well as the validity and reliability of NRQ-INA to measure social strain among the caregivers of dementia was beyond the aim and scope of this study. Therefore, further study with a larger sample size is needed to investigate the concurrent validity of NRQ-INA as a predictor of social strain, particularly in people with dementia and their caregivers.

## 5. Conclusions

In conclusion, the validity and reliability of NRQ-INA were acceptable to measure the social strain among PWD in Indonesia. Further study with a larger sample size is needed to investigate the concurrent validity of NRQ-INA as a predictor of social strain, particularly in people with dementia and their caregivers.

## Figures and Tables

**Table 1:** Baseline characteristics of research subjects.

Characteristics	*n*	%
Gender		
Male	39	65
Female	21	35
Age		
>65 years old	40	66.7
<65 years old	20	33.3
Dementia type		
Alzheimer’s dementia	12	20
Vascular dementia	35	58.3
Mixed-type dementia	9	15
Other dementia		6.7
SOP-related dementia	1	1.675
Post-TBI-related dementia	1	1.675
Subcortical dementia	1	1.675
Epilepsy-related Dementia	1	1.675

SOP: space-occupying process; TBI: traumatic brain injury.

**Table 2:** NRQ-INA validity test results for each item of the questionnaire.

Item	*r*	*p* Value	Interpretation (*r* > 0.35)
Spouse			
Q1	0.858	<0.001 **	Strongly Valid
Q2	0.805	<0.001 **	Strongly Valid
Q3	0.834	<0.001 **	Strongly Valid
Q4	0.822	<0.001 **	Strongly Valid
Friend			
Q1	0.837	<0.001 **	Strongly Valid
Q2	0.815	<0.001 **	Strongly Valid
Q3	0.907	<0.001 **	Strongly Valid
Q4	0.902	<0.001 **	Strongly Valid
Family member			
Q1	0.837	<0.001 **	Strongly Valid
Q2	0.837	<0.001 **	Strongly Valid
Q3	0.890	<0.001 **	Strongly Valid
Q4	0.855	<0.001 **	Strongly Valid

NRQ-INA: Negative Relationship Quality questionnaire-Indonesia version. ** *p* < 0.001.

**Table 3:** KMO and factor analysis of items in each subscale of NRQ-INA questionnaire.

Item	KMO	Factor1 (Rotation Matrix)
Spouse	0.840 **	
How often do they criticize you?		0.926
How often do they make too many demands on you?		0.869
How often do they let you down when you are counting on them?		0.924
How often do they get on your nerves?		0.900
Friend	0.859 **	
How often do they criticize you?		0.921
How often do they make too many demands on you?		0.874
How often do they let you down when you are counting on them?		0.951
How often do they get on your nerves?		0.948
Family member	0.826 **	
How often do they criticize you?		0.911
How often do they make too many demands on you?		0.911
How often do they let you down when you are counting on them?		0.939
How often do they get on your nerves?		0.919

NRQ-INA: Negative Relationship Quality questionnaire-Indonesia version; KMO: Kaiser–Meyer–Olkin. ** *p* < 0.001.

**Table 4:** NRQ-INA reliability test results (internal consistency reliability) for each item of the questionnaire.

Item	Cronbach’s Alpha	Interpretation (Cronbach’s Alpha > 0.7)
Spouse		
Q1	0.895	Reliable
Q2	0.911	Reliable
Q3	0.901	Reliable
Q4	0.907	Reliable
Friend		
Q1	0.934	Reliable
Q2	0.940	Reliable
Q3	0.910	Reliable
Q4	0.912	Reliable
Family member		
Q1	0.923	Reliable
Q2	0.925	Reliable
Q3	0.909	Reliable
Q4	0.918	Reliable

**Table 5:** Results of the test–retest reliability (external consistency) of NRQ-INA.

Item Number	Pearson Coefficient of Correlation (between Test and Retest)	*p* Value	Interpretation
Spouse			
Q1	0.750	<0.001 **	Reliable
Q2	0.776	<0.001 **	Reliable
Q3	0.878	<0.001 **	Reliable
Q4	0.836	<0.001 **	Reliable
Friend			
Q1	0.899	<0.001 **	Reliable
Q2	0.892	<0.001 **	Reliable
Q3	0.842	<0.001 **	Reliable
Q4	0.878	<0.001 **	Reliable
Family member			
Q1	0.875	<0.001 **	Reliable
Q2	0.863	<0.001 **	Reliable
Q3	0.817	<0.001 **	Reliable
Q4	0.928	<0.001 **	Reliable

NRQ-INA: Negative Relationship Quality questionnaire-Indonesia version. ** *p* < 0.001.

**Table 6:** The total score of NRQ-INA based on gender, age, and dementia type.

Variables	NRQ-INA Total Score (Median)	*p* Value
Gender		0.965
Male	39.00	
Female	39.75	
Age		0.867
<65	38.75	
≥65	39.25	
Dementia type		0.709
Alzheimer’s dementia	35.00	
Vascular dementia	40.00	
Mixed dementia	37.50	
Other dementia	38.50	

## Data Availability

The data presented in this study are openly available in: https://zenodo.org/record/7080885#.YyLB-S8Rqu4 (accessed on 4 August 2022).
